# Toxicity profiling of the ethanolic extract of *Citrullus lanatus* seed in rats: behavioral, biochemical and histopathological aspects

**DOI:** 10.1042/BSR20202345

**Published:** 2021-01-08

**Authors:** Sateesh Belemkar, Parshuram Nivrutti Shendge

**Affiliations:** 1 Department of Pharmacology, Shobhaben Pratapbhai Patel School of Pharmacy and Technology Management, SVKM’S NMIMS, V.L. Mehta Road, Vile Parle (W), Mumbai 400 056, Maharashtra, India; 2 Department of Pharmacology, School of Pharmacy and Technology Management, SVKM’S NMIMS, Shirpur 425 405, Maharashtra, India

**Keywords:** acute toxicity, subacute toxicity, body weight, wistar rat

## Abstract

*Citrullus lanatus* (Cucurbitaceae) is conventionally used for the treatment of urinary tract infection, renal stones, hypertension, diabetes and diarrhoea. Current study evaluates acute and 28 days repeated toxicity ethanolic extract of *C. lanatus* seed (EECLS) in Wistar rats to measure its safety profile. The single dose (2000 mg/kg BW) of EECLS was administered while in 28 days repeated study 250, 500 and 1000 mg/kg BW were administered orally in rats. Parameters such as biochemical, haematological and histopathological were analysed in subacute toxicity study. During study, no apparent sign of toxicity, behavioural changes and mortality were detected in acutely exposed animals. In 28 days repeated toxicity study, rats did not show significant changes in behaviour, gross pathology, body weight, biochemical and haematological parameters. Abridged serum glucose and cholesterol levels during the study designate their roles in treatment of hyperglycaemic and hyperlipidaemic conditions. No significant difference was observed in histopathology of liver and kidneys of treated rats. The current investigation demonstrated that EECLS is non-toxic below 1000 mg/kg BW and provides protection to some body organs. The data propose that LD_50_ of EECLS was greater than 2000 mg/kg BW and the no observed adverse effect level (NOAEL) of EECLS was at the dose of 1000 mg/kg in rats. Taken together, our finding suggests that, EECLS is safe and provides some protection to body organs; also, its extract can be used for further preclinical and clinical evaluation for its therapeutic activity.

## Introduction

Since ancient times, plants have proved their effectiveness in treatment of different illnesses. Due to their low cost and availability, formulations prepared from natural origin are preferred for medicinal uses. Despite their efficacy, very less evidence is available for toxicity associated with plant-based products. Data for possible toxicity of plant(s) are not available which makes it necessary to evaluate medicinal plant(s) for their possible toxicity. The toxicity studies performed on plants extract will help to increase confidence of researchers in safe use of natural substances for human consumption [1–4].

*Citrullus lanatus* also known as watermelon is an annual herb belonging to Cucurbitaceae family cultivated in India, China, Iran, Turkey and South Africa. This plant consists of long stems with curly tendrils and hairy leaves which are 3–19 cm long with 3–5 lobes. It is monoecious plant where male flowers have 1.2–4.5 cm long pedicels while female flower is pale green in colour and 1–2.5 cm long. Fruits are 1.5–20 cm in diameter, spherical in shape, dark green in colour with long stalk. The wild form of fruits has yellow or green pulp and cultivated pulp is dark red in colour. The seeds of the plant are yellow to dull brown or black in colour and ovate in shape [5].

*C. lanatus* fruit contains 92% water and approximately 6% sugar. Fruits are good source of vitamins and minerals. The dried seeds of *C. lanatus* contains proteins (28.3 g), fats (47.4 g), carbohydrates (15.3 g) and calcium (54 mg) per 100 g. Active constituents such as lycopene, β-carotene, glycoprotein-vivilin, 2-dodecyclobutanon, terpene, xanthophylls etc are also present in the seed [6–8]. Extracts of the seed also possess pharmacological actions such as anti-inflammatory, anti-ulcer, antimicrobial, hepatoprotective and antioxidant activity [9]. The efficacy of watermelon fruit has been studied widely but data about safety profile of extract of *C. lanatus* seed are still scarce. The current investigation is performed to evaluate the acute and 28 days repeated toxicity of ethanolic extract of *C. lanatus* seed (EECLS) in Wistar rats.

## Materials and methods

### Plant material and extraction

*C. lanatus* fruits were collected from Shirpur area (Maharashtra, India) (latitude 21°21′21.7′′ north, longitude 74°53′02.0′′ east and altitude 252 metres) in May 2019. The fruit was identified and authenticated from the Agharkar Research Institute, Pune and the voucher specimen was deposited in the institute’s herbarium department for future reference. The dried seeds were powdered in a grinder and extracted by maceration process with ethanol for 48 h. After 48 h, the solution was filtered using Whatman N°1 filter paper (11 μm) and the filtrate was concentrated using a vacuum rotary evaporator and dried by means of a freeze dryer. The extract was stored in well-closed container at a cool place. The extract was administered orally, using gavage at the respective doses for the acute and subacute toxicity studies.

### Phytochemical screening

Phytoconstituents present in EECLS were detected by employing various chemical tests. The main bioactive groups (alkaloids, tannins, saponins, flavonoids, cardiac glycosides and polyphenols) were identified using different standard methods [10].

#### Test for alkaloids

EECLS was stirred with 1% HCl (5 ml) on water bath and filtered. The filtrate (1 ml) treated with Mayer’s reagent and another 1 ml of filtrate treated was with Dragendorff reagent. Precipitation or turbidity appearance indicated presence of alkaloid in extract.

#### Test for tannins

Extract (0.5 ml) was mixed with ferric chloride (FeCl_3_) and appearance of blue-black precipitate specified presence of tannin.

#### Test for saponins

EECLS (1 ml) was mixed with water and mixture was shaken, appearance of persisted frothing demonstrated presence of saponins.

#### Test for flavonoids

EECLS (2 ml) was mixed with concentrated HCl (2 ml) and magnesium turnings were added to it. Appearance of crimson red/pink-scarlet colour indicated presence of flavonoid.

#### Test for cardiac glycosides

The plant extract (2 ml) was mixed with glacial acetic acid (1 ml), FeCl_3_ (1 ml) and concentrated H_2_SO_4_ (1 ml). Green-blue colour showed the presence of cardiac glycosides.

#### Test for polyphenols

A total of 2 ml of EECLS was kept for 30 min in water bath and 1 ml of 1% FeCl_3_ along with potassium ferrocyanide (1 ml) was added, mixed and filtered. Formation of blue-green colour indicated presence of polyphenols.

### Experimental animals

Wistar rats (150–200 g body weight) were housed in a standard temperature (22 ± 3°C) and humidity (45–55%) in institutional animal house facility in SVKM’s NMIMS SPTM, Shirpur campus, Maharashtra, India. During the study, 12-h light/dark cycle was followed. Animals were allowed free access to food and water. Standard pellet diet was provided during the study. The study was conducted in accordance with the Basic and Clinical Pharmacology and Toxicology policy for experimental and clinical studies [11]. All the procedures performed during the study were approved by local animal ethics committee constituted by SVKM’s NMIMS SPTM, Shirpur campus, Maharashtra, India (Protocol number: SPTM-IAEC/DEC-17/02/06).

### Acute oral toxicity study

The acute toxicity study of EECLS was implemented as per Organization for Economic Cooperation and Development (OECD) guideline 423. Wistar rats (150–190 g) were divided into two groups each comprising three animals (female). First group acted as a control which received purified drinking water and second served as treatment group receiving EECLS (single dose of 2000 mg/kg BW) via oral route. Extract was dissolved in purified water to prepare stock solution and 1 ml of stock solution administered using oral gavage. Animals were closely monitored for various behavioural parameters first 24 h after dosing and once daily for 14 days. Body weight, food and water intake were documented. Special functional observational battery was established to evaluate animal behavioural response in open-field and home-cage conditions [12,13].

After 14 days, animals were killed by the process of decapitation and various organs removed, weighed to calculate relative organ weight. The relative organ weight was calculated using formula (weight of organ/body weight of rat on day of killing) × 100% [14].

### Subacute oral toxicity study

Subacute toxicity study was performed as per the OECD Test Guidelines 407 [15]. Animals were divided into four groups consisting of ten animals (five males and five females) in each group. Group I served as vehicle control and received purified drinking water by oral route. Remaining three groups, i.e. group II, III and IV served as treatment group and received 250, 500 and 1000 mg/kg of extract respectively dissolved in purified water by oral route for 28 days.

Body weight, food and water intake were observed and recorded daily. Other parameters such as changes in behaviour, mortality and adverse effects were recorded. After 28 days, blood samples were removed by retro-orbital method and collected in plain and heparinised bottles. Non-heparinised blood samples centrifuged at 3000 rpm for 10 min in multispin centrifuge and separated serum was used to evaluate biochemical parameters associated with liver functions (serum aspartate aminotransferase (AST), alkaline phosphatase (ALP) and alanine aminotransferase (ALT)), kidney function (serum total protein, urea and creatinine) and lipid profile (total cholesterol and triglycerides) were analysed using commercially available kits on Erba chem-7 analyser.

Heparinised blood samples used to evaluate as Packed cell volume (PCV), White blood count (WBC), Neutrophils (NPs), Lymphocytes (LCs), Monocytes (MCs), Eosinophils (EPs), Haemoglobin (HB), Platelets (PLs), Red blood cell (RBC) using Exigo EOS-Vet cell counter [16]. Organs such as kidney and liver were collected and fixed in 10% buffered formalin for histopathology. Subsequently they were submitted for routine histopathological examination.

### Statistical analysis

The results were analysed using one-way analysis of variance (ANOVA), followed by Dunnett’s test. Data are expressed as mean ± SEM and the differences between groups were statistically significant when *P*<0.05.

## Results

### Phytochemical screening of the plant extracts

The phytochemical testing of EECLS revealed presence of saponins and glycosides (Table 1).

**Table 1:** Phytochemical profile of EECLS

Tests	P	T	St	Ta	F	Al	S	Gl
**Plant Extract**	-	-	-	-	-	-	+	+

The sign (+) indicates the presence of the compounds and (-) the absence.

Abbreviations: Al, alkaloids; Gl, glycosides; F, flavonoids; P, polyphenols; S, saponins; St, steroids; T, terpenoids; Ta, tannin.

### Acute toxicity study

**Figure 1:**
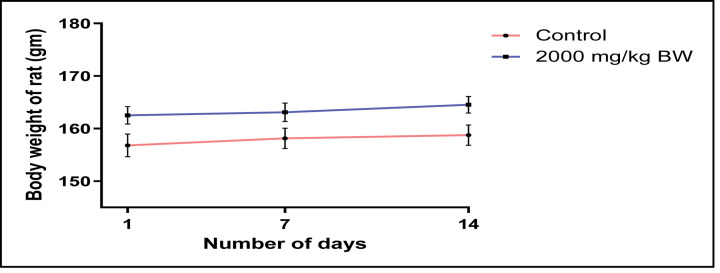
Body weight of control and EECLS-treated rats at a single dosage of 2000 mg/kg No significant weight gain was observed after acute treatment of EECLS during 14 days.

**Figure 2:**
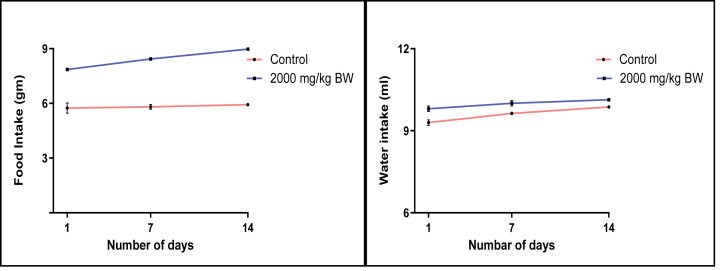
Food and water intake of control and EECLS -reated rats at a single dosage of 2000 mg/kg BW Food and water intake after acute administration of EECLS showed no significant changes during 14 days observation.

During and after the study, no mortality was observed in treated animal. No behavioural changes were observed in animals treated with EECLS (Table 2). The body weight, food and water intake of animals belonging to treated group did not show any significant variation (Figures 1 and 2). After 14 days, animals were killed and various vital organs such as liver, lung, spleen, heart and kidney observed for any damage. No damage or necrosis was observed in vital organs. Acute treatment with EECLS did not produce any changes in absolute and relative organ weight of animal (Table 3). Functional behavioural battery parameter evaluation for first 24 hours (Table 4) and thereafter once daily for 14 days (Table 5) showed no changes in behaviour of animal. Necropsy of the vital organs also showed no changes in structure (Table 6). As no mortality was observed during the study, LD_50_ of EECLS was considered as 2000 mg/kg BW for experimental rats in both sexes. Histopathological evaluation of kidney and liver cells after acute treatment with extract displayed no changes in tissue architecture (Figures 6A,B and 7A,B).

**Table 2:** Cageside observations for 14 days of control and EECLS-treated rats at a single dosage of 2000 mg/kg BW

Parameters	Observations		
	On day 0	On day 7	On day 14
Condition of the fur	Normal	Normal	Normal
Change in skin	No effect	No effect	No effect
Body posture	Normal	Normal	Normal
Eye colour	No change	No change	No change
Diarrhoea	Not present	Not present	Not present
Respiration	Normal	Normal	Normal
Sedation	No effect	No effect	No effect
Drowsiness	Not present	Not present	Not present
Coma	Not present	Not present	Not present
Mortality	Alive	Alive	Alive

**Table 3:** Absolute and relative organ weight of control and EECLS-treated rats at a single dosage of 2000 mg/kg BW

Organ	Absolute organ weight (g)		Relative organ weight (%)	
	Control	2000 mg/kg BW	Control	2000 mg/kg BW
Liver	7.6 ± 0.3	7.2 ± 0.1	4.0 ± 0.2	4. 2 ± 0.1
Heart	0.5 ± 0.1	0.4 ± 0.1	0.4 ± 0.2	0.7 ± 0.1
Kidney	1.0 ± 0.1	0.9 ± 0.1	0.8 ± 0.1	0.9 ± 0.1
Lung	1.2 ± 0.1	1.5 ± 0.1	0. 9 ± 0.2	1.1 ± 0.2
Intestine	6.4 ± 0.1	6.8 ± 0.2	5.7 ± 0.1	5.9 ± 0.1

Data indicate mean ± SEM, *n*=3. There are no significant (*P*<0.05) differences between the control and MELSF-treated rats in their absolute and relative organ weights.

**Table 4:** Functional behavioural battery parameters on the day of dosing of control and EECLS-treated rats at a single dosage of 2000 mg/kg BW

Groups		Control							2000 mg/kg BW						
Parameters	Normal score	30 min	1 h	2 h	4 h	8 h	12 h	24 h	30 min	1 h	2 h	4 h	8 h	12 h	24 h
Awareness															
**Alertness**	4	4	4	4	4	4	4	4	3	4	4	4	4	4	4
Visual placing	4	4	4	4	4	4	4	4	4	4	4	4	4	4	4
Passivity	0	0	0	0	0	0	0	0	0	0	0	0	0	0	0
Stereotypy	0	0	0	0	0	0	0	0	0	0	0	0	0	0	0
**Mood**															
Grooming	4	4	4	4	4	4	4	4	4	4	4	4	4	4	4
Vocalisation	0	0	0	0	0	0	0	0	0	0	0	0	0	0	0
Restlessness	0	0	0	0	0	0	0	0	0	0	0	0	0	0	0
Irritability	0	0	0	0	0	0	0	0	1	1	0	0	0	0	0
Fearfulness	0	0	0	0	0	0	0	0	0	0	0	0	0	0	0
**Motor activity**															
Reactivity	4	4	4	4	4	4	4	4	3	3	4	4	4	4	4
Spont. activity	4	4	4	4	4	4	4	4	4	4	4	4	4	4	4
Touch response	4	4	4	4	4	4	4	4	4	4	4	4	4	4	4
Pain response	4	4	4	4	4	4	4	4	4	4	4	4	4	4	4
**CNS excitation**															
Startle response	0	0	0	0	0	0	0	0	0	0	0	0	0	0	0
Straub response	0	0	0	0	0	0	0	0	0	0	0	0	0	0	0
Tremors	0	0	0	0	0	0	0	0	0	0	0	0	0	0	0
Twitches	0	0	0	0	0	0	0	0	0	0	0	0	0	0	0
Convulsion	0	0	0	0	0	0	0	0	0	0	0	0	0	0	0
**Posture**															
Body posture	4	4	4	4	4	4	4	4	3	4	4	4	4	4	4
Limb position	4	4	4	4	4	4	4	4	4	4	4	4	4	4	4
**Motor incoord.**															
Staggering gait	0	0	0	0	0	0	0	0	0	0	0	0	0	0	0
Abnormal gait	0	0	0	0	0	0	0	0	1	0	0	0	0	0	0
Righting reflex	0	0	0	0	0	0	0	0	0	0	0	0	0	0	0
**Muscle tone**															
Limb tone	4	4	4	4	4	4	4	4	4	4	4	4	4	4	4
Grip strength	4	4	4	4	4	4	4	4	3	4	4	4	4	4	4
Body sag	0	0	0	0	0	0	0	0	0	0	0	0	0	0	0
Body tone	4	4	4	4	4	4	4	4	4	4	4	4	4	4	4
Abdominal tone	4	4	4	4	4	4	4	4	4	4	4	4	4	4	4
**Reflexes**															
Pinne	4	4	4	4	4	4	4	4	4	4	4	4	4	4	4
Corneal	4	4	4	4	4	4	4	4	4	4	4	4	4	4	4
IFR	4	4	4	4	4	4	4	4	4	4	4	4	4	4	4
**Autonomic**															
Writhing	0	0	0	0	0	0	0	0	0	0	0	0	0	0	0
Pupil size	4	4	4	4	4	4	4	4	4	4	4	4	4	4	4
Palpeb. opening	4	4	4	4	4	4	4	4	4	4	4	4	4	4	4
Exophthalmos	0	0	0	0	0	0	0	0	0	0	0	0	0	0	0
Urination	0	0	0	0	0	0	0	0	0	0	0	0	0	0	0
Salivation	0	0	0	0	0	0	0	0	0	0	0	0	0	0	0
Pilo erection	0	0	0	0	0	0	0	0	0	0	0	0	0	0	0
Hypothermia	0	0	0	0	0	0	0	0	0	0	0	0	0	0	0
Skin colour	4	4	4	4	4	4	4	4	4	4	4	4	4	4	4
Heart rate	4	4	4	4	4	4	4	4	4	4	4	4	4	4	4
Resp. rate	4	4	4	4	4	4	4	4	4	4	4	4	4	4	4
**Miscellaneous**															
Lacrimation	0	0	0	0	0	0	0	0	0	0	0	0	0	0	0
Dead															
No. Acute	0	0	0	0	0	0	0	0	0	0	0	0	0	0	0
No. Delayed	0	0	0	0	0	0	0	0	0	0	0	0	0	0	0

All animals were kept under observation for 24 h after administration of EECLS to evaluate behavioural changes. No severe behavioural changes were observed in functional behavioural battery parameters during first 24 h.

Scores were given on the basis of change in behaviour with respect to normal scores.

**Table 5:** Functional behavioural battery parameters observed once a week of control and EECLS-treated rats at a single dosage of 2000 mg/kg BW

Groups		Control			2000 mg/kg BW		
Parameters	Normal score	Day 1	Day 7	Day 14	Day 1	Day 7	Day 14
**Awareness**							
Alertness	4	4	4	4	4	4	4
Visual placing	4	4	4	4	4	4	4
Passivity	0	0	0	0	0	0	0
Stereotypy	0	0	0	0	0	0	0
**Mood**							
Grooming	4	4	4	4	4	4	4
Vocalisation	0	0	0	0	0	0	0
Restlessness	0	0	0	0	0	0	0
Irritability	0	0	0	0	0	0	0
Fearfullness	0	0	0	0	0	0	0
**Motor activity**							
Reactivity	4	4	4	4	4	4	4
Spont. activity	4	4	4	4	4	4	4
Touch response	4	4	4	4	4	4	4
Pain response	4	4	4	4	4	4	4
**CNS excitation**							
Startle response	0	0	0	0	0	0	0
Straub response	0	0	0	0	0	0	0
Tremors	0	0	0	0	0	0	0
Twitches	0	0	0	0	0	0	0
Convulsion	0	0	0	0	0	0	0
**Posture**							
Body posture	4	4	4	4	4	4	4
Limb position	4	4	4	4	4	4	4
**Motor incoord.**							
Staggering gait	0	0	0	0	0	0	0
Abnormal gait	0	0	0	0	0	0	0
Righting reflex	0	0	0	0	0	0	0
**Muscle tone**							
Limb tone	4	4	4	4	4	4	4
Grip strength	4	4	4	4	4	4	4
Body sag	0	0	0	0	0	0	0
Body tone	4	4	4	4	4	4	4
Abdominal tone	4	4	4	4	4	4	4
**Reflexes**							
Pinne	4	4	4	4	4	4	4
Corneal	4	4	4	4	4	4	4
IFR	4	4	4	4	4	4	4
**Autonomic**							
Writhing	0	0	0	0	0	0	0
Pupil size	4	4	4	4	4	4	4
Palpeb. opening	4	4	4	4	4	4	4
Exophthelmes	0	0	0	0	0	0	0
Urination	0	0	0	0	0	0	0
Salivation	0	0	0	0	0	0	0
Pilo erection	0	0	0	0	0	0	0
Hypothermia	0	0	0	0	0	0	0
Skin colour	4	4	4	4	4	4	4
Heart rate	4	4	4	4	4	4	4
Resp. rate	4	4	4	4	4	4	4
**Miscellaneous**							
Lacrimation	0	0	0	0	0	0	0
**Dead**							
No. Acute	0	0	0	0	0	0	0
No. Delayed	0	0	0	0	0	0	0

No behavioural changes were observed in functional behavioural battery parameters after administration of EECLS.

Scores were given on the basis of change in behaviour with respect to normal scores.

### Subacute toxicity

#### Effect of EECLS on body weights and mortality

**Figure 3:**
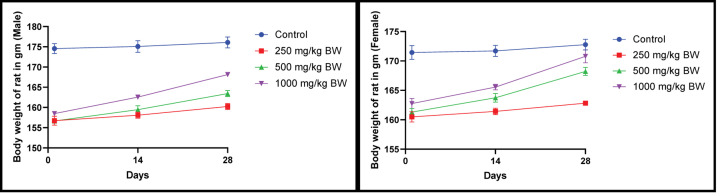
Body weight of rat after administration of 250, 500 and 1000 mg/kg EECLS for 28 days Body weight of male and female rats did not show any stastistical alteration during 28 days of study. Dose-dependent increase in body weight observed for all doses. Values expressed as ± SEM, one-way ANOVA followed by Dunnett’s test, (*n*=5 animals/group). Differences between groups were considered to be significant when *P*<0.05.

After 28 days, no treatment-related apparent toxicity and mortality were detected in animals. The mean value of gain in body weight of treated and control groups showed that extract showed no significant changes on body weight (Figure 3).

#### Effect of EECLS on food intake and water consumption in rats

**Figure 4:**
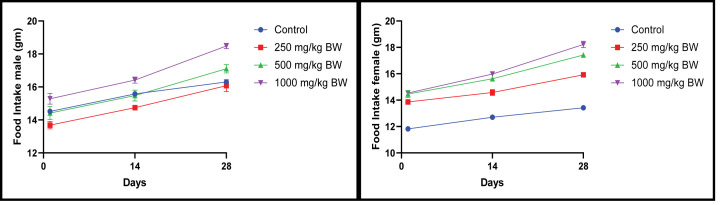
Food intake of rat after administration of 250, 500 and 1000 mg/kg EECLS for 28 days Dose-dependent increase in food intake was noticed in all groups during the study but no significant changes appeared due to food intake. Values expressed as ± SEM, one-way ANOVA followed by Dunnett’s test (*n*=5 animals/group). Differences between groups were considered to be significant when *P*<0.05.

**Figure 5:**
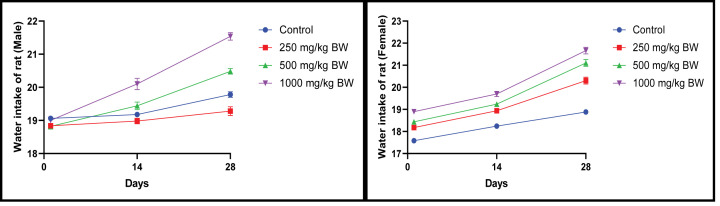
Water intake of rat after administration of 250, 500 and 1000 mg/kg EECLS for 28 days Water intake of male and female rats in all group remained within significant limit during 28-day study period. Values expressed as ± SEM, one-way ANOVA followed by Dunnett’s test (*n*=5 animals/group). Differences between groups were considered to be significant when *P*<0.05.

Daily food and water consumption was recorded but no statistical alteration observed which indicate extract does not have any obvious effect on food and water intake (Figures 4 and 5).

#### Effect of EECLS on haematological parameters

The relative blood parameters such as monocytes, lymphocytes, RBS, WBC etc were not changed statistically in EECLS-treated rats when compared with normal control group (Table 7).

#### Effect of EECLS on serum marker enzymes during the 28-day treatment

The levels of ALP, AST and ALT were not changed significantly after treatment with EECLS indicating that extract did not exert any effect on normal level of these serum marker enzymes (Table 8).

#### Effect of EECLS on serum total protein, albumin, urea and creatinine

Serum total protein, albumin, urea, uric acid and creatinine levels remains statistically unchanged when compared with respective normal control group. Also, no changes in urea and uric acid levels were observed after treatment of EECLS (Table 8).

#### Effect of EECLS on lipid profile and glucose level

The levels of serum triglycerides, total cholesterol and glucose remain statistically unaltered after treatment of 28 days (Table 8).

#### Effect of EECLS on histopathology

**Figure 6:**
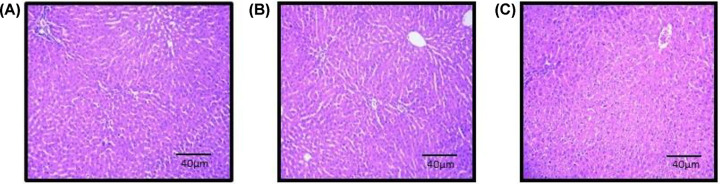
Histopathology of Liver Photometry of liver of control group (**A**), group treated with 2000 mg/kg (**B**) (acute toxicity study) and group treated with 1000 mg/kg (**C**) (repeated toxicity study). No histopathological alteration was observed in liver of 2000 mg/kg (acute toxicity) and 1000 mg/kg (repeated toxicity) of EECLS-treated rat when compared with control group.

**Figure 7:**
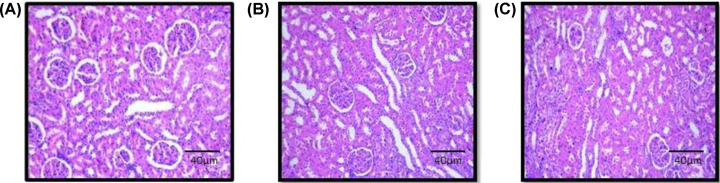
Histopathology of Kidney Photometry of kidney of control group (**A**), group treated with 2000 mg/kg (**B**) (acute toxicity study) and group treated with 1000 mg/kg (**C**) (repeated toxicity study). Histopathological assessment of kidney in acute and subacute toxicity study showed no pathological changes after 28 days treatment.

Histopathological analysis of liver and kidney of treated group showed normal tissue architecture and absence of pathological lesions when compared with normal control. Also, microscopic evaluation of treated rats indicated no abnormalities in texture or colour of organ (Figures 6A,C and 7A,C).

## Discussion

Herbal medicines and formulations prepared from the plants have been safe and effective owing to their insignificant side effects. The safety assumptions associated with herbal medicine may have prejudiced the rural population to make hefty use of them. In developing countries where rural population is large, these formulations are administered via oral route for extensive period without proper consultation and dosage monitoring, which might facilitate lack of awareness towards toxic effects associated with them. Therefore, scientifically proven data towards oral toxicity of herbal medicines are much desired which not only help to recognise safe doses for further use but also disclose the different clinical signs and symptoms associated with herbal agent under examination. Various pharmacological actions of *C. lanataus* have been proved but detailed knowledge regarding toxicity of the plant is lacking. Hence, to estimate the safety data of *C. lanatus* seed the current study was commenced [17–19].

The preliminary step in the screening of pharmacological activity is the evaluation of toxic features of the plant extract or isolated compounds. The acute toxicity offers information which can be used to classify, label and calculate the dose of novel compound in animal studies. In current study, the acute dosing of EECLS, i.e. 2000 mg/kg BW is found to be safe as no mortality or any significant change in body weight, food consumption and water intake observed. Toxic substance exerts their effects on major systemic organs such as kidney, heart, liver, lung and heart. The necropsy of internal organs showed no changes in size and shape when compared with respective control group (Table 6). The histoarchitecture assessment of kidney and liver cells did not display any variations after the acute treatment of EECLS (Figures 6A,B and 7A,B). As all the animals used in the study were survived until forced euthanasia, therefore an acute LD_50_ of EECLS in rats was determined to be greater than 2000 mg/kg BW. Thus, the design of our 28 days repeated toxicity study was based on LD_50_ dose and three different doses (250, 500 and 1000 mg/kg) representing low, medium and high dose, respectively, were analysed in order to determine a no observed adverse effect levels (NOAELs) in rats. To our acquaintance, till date there is no systematic research regarding subacute toxicity of EECLS to support clinical drug safety. Therefore, in current investigation, we performed a comprehensive experiment to evaluate the toxicity and safety of EECLS in Wistar rats.

**Table 6:** Gross necropsy study of vital organs of control and EECLS-treated rats (2000 mg/kg BW)

Vital organs	Control group	2000 mg/kg BW
Liver	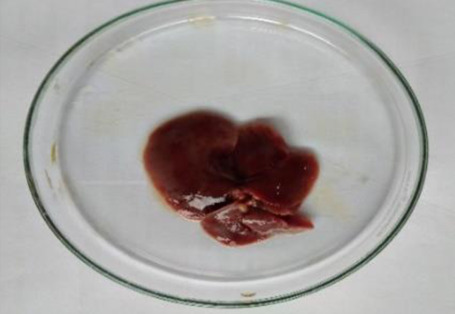	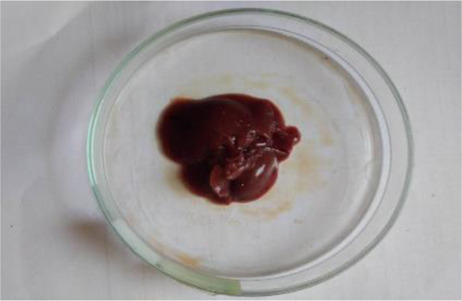
Heart	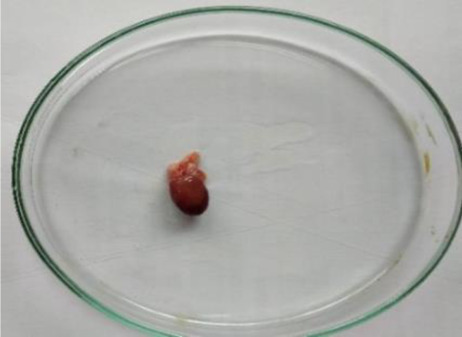	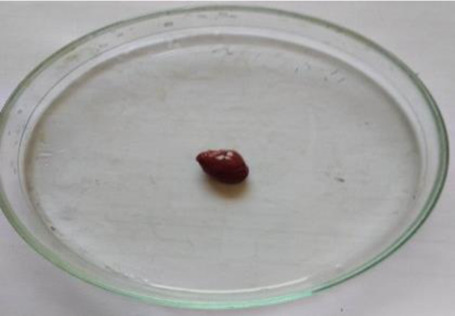
Kidney	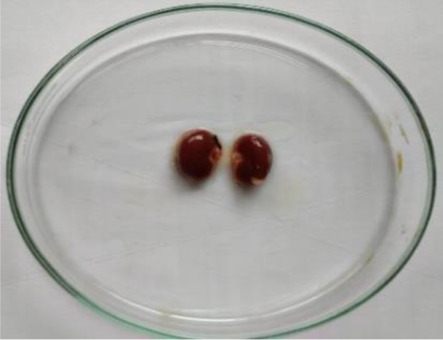	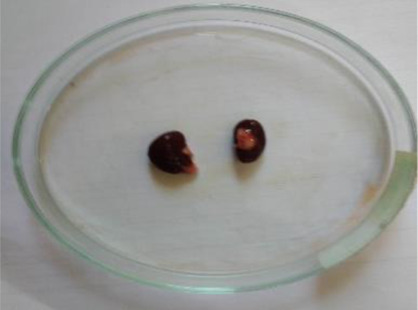
Lungs	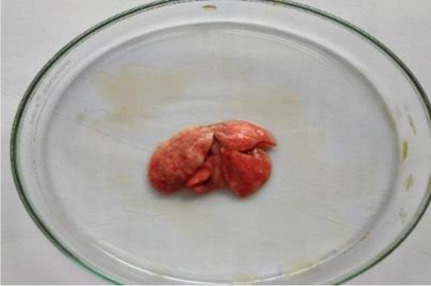	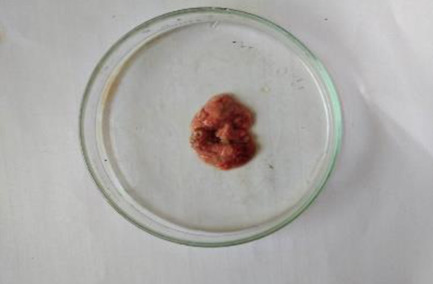
Small intestine	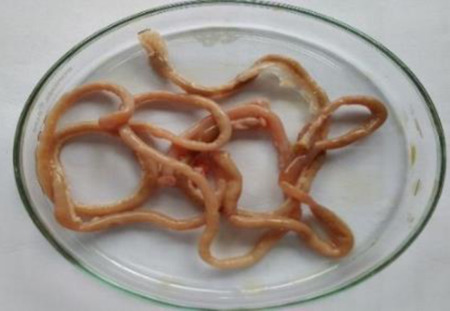	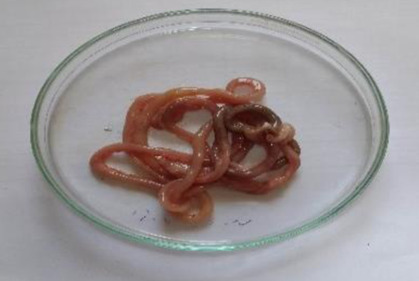

The gross necropsy of vital organs showed that no effect of EECLS was observed and all organs structure remain unaltered.

**Table 7:** Haematological parameters of rats after administration of 250, 500 and 1000 mg/kg EECLS for 28 days

Parameters	Male				Female			
	Control	250 mg/kg	500 mg/kg	1000 mg/kg	Control	250 mg/kg	500 mg/kg	1000 mg/kg
**WBC (×10^3^/μl)**	8.6 ± 1.10	9.4 ± 1.01	9.3 ± 1.09	9.7 ± 1.10	8.9 ± 1.35	11.7 ± 1.20	10.4 ± 1.82	10.5 ± 1.32
**RBC (×10^6^/μl)**	8.3 ± 0.12	8.9 ± 0.13	8.8 ± 0.16	8.9 ± 0.09	8.0 ± 0.21	9.4 ± 0.19	9.1 ± 0.09	9.2 ± 0.08
**Hb (g/dl)**	16.3 ± 0.31	16.4 ± 0.24	16.3 ± 0.07	16.7 ± 0.10	16.4 ± 0.26	16.7 ± 0.12	16.3 ± 0.18	16.4 ± 0.14
**NPs (%)**	10.8 ± 1.35	9.9 ± 1.86	10.5 ± 1.45	10.9 ± 1.84	10.3 ± 1.10	10.1 ± 1.14	10.0 ± 1.36	10.5 ± 1.22
**LCs (%)**	82.57 ± 1.16	86.34 ± 1.24	89.52 ± 1.19	89.87 ± 1.58	85.41 ± 1.04	82.53 ± 1.44	86.65 ± 0.89	86.27 ± 1.48
**MCs (%)**	1.8 ± 0.12	1.8 ± 0.07	1.9 ± 0.19	1.8 ± 0.20	1.8 ± 0.21	2.2 ± 0.11	2.3 ± 0.31	2.1 ± 0.24
**EPs (%)**	1.0 ± 0.06	0.7 ± 0.15	1.0 ± 0.07	0.9 ± 0.14	1.2 ± 0.31	1.5 ± 0.08	1.9 ± 0.07	2.1 ± 0.09
**Basophils (%)**	0.8 ± 0.07	0.8 ± 0.09	0.9 ± 0.08	1.0 ± 0.06	0.8 ± 0.21	0.7 ± 0.24	1.0 ± 0.09	1.1 ± 0.06
**PLs (×10^6^/μl)**	736.41 ± 20.13	850.32 ± 12.13	875.36 ± 13.11	894.15 ± 16.10	784.15 ± 18.54	890.32 ± 13.15	884.18 ± 8.14	883.21 ± 19.46
**MCV (fl)**	61.5 ± 0.65	61.4 ± 0.75	63.7 ± 0.84	65.8 ± 1.19	58.74 ± 0.64	63.73 ± 1.26	65.93 ± 1.29	67.45 ± 1.64
**MCH (pg)**	19.6 ± 0.14	19.9 ± 0.31	20.3 ± 0.18	20.2 ± 0.21	19.8 ± 0.28	19.6 ± 0.24	20.4 ± 0.18	21.7 ± 0.09
**MCHC (g/dl)**	31.82 ± 0.27	32.35 ± 0.29	33.42 ± 0.24	35.16 ± 0.28	32.45 ± 0.25	33.75 ± 0.16	34.62 ± 0.34	36.37 ± 0.423

Values expressed as ± SEM, one-way ANOVA followed by Dunnett’s test (*n*=5 animals/group). Differences between groups were considered to be significant when *P*<0.05.

**Table 8:** Biochemical parameters of rat after administration of 250, 500 and 1000 mg/kg EECLS for 28 days

Parameters	Male				Female			
	Control	250 mg/kg	500 mg/kg	1000 mg/kg	Control	250 mg/kg	500 mg/kg	1000 mg/kg
**Urea (mg/dl)**	14.2 ± 1.23	14.6 ± 0.34	14.9 ± 0.38	15.7 ± 0.21	16.3 ± 1.16	15.8 ± 1.13	14.8 ± 0.94	15.2 ± 0.84
**Uric acid (mg/dl)**	2.1 ± 0.06	2.12± 0.14	2.24 ± 0.11	1.86 ± 0.06	2.34 ± 0.05	2.08 ± 0.07	2.23 ± 0.08	1.98 ± 0.13
**Total protein (g/dl)**	6.3 ± 0.18	6.5 ± 0.08	6.4 ± 0.06	6.4 ± 0.07	6.2 ± 0.16	6.3 ± 0.18	6.4 ± 0.30	6.5 ± 0.24
**Creatine (mg/dl)**	0.6 ± 0.05	0.62 ± 0.09	0.66 ± 0.05	0.55 ± 0.09	0.71 ± 0.04	0.84 ± 0.08	0.82 ± 0.25	0.74 ± 0.27
**Albumin (g/dl)**	3.21 ± 0.08	3.23 ± 0.09	3.54 ± 0.06	3.49 ± 0.07	3.42 ± 0.11	3.21 ± 0.09	3.34 ± 0.07	3.23 ± 0.08
**Glucose (mg/dl)**	101.41 ± 3.49	104.23 ± 3.97	100.17 ± 5.41	97.43 ± 4.15	100.8 ± 6.29	113.47 ± 7.54	108.42 ± 3.87	104.62 ± 3.43
**ALT (SGPT) (U/l)**	55.60 ± 1.06	49.38 ± 2.19	49.62 ± 1.35	49.53 ± 1.67	57.24 ± 2.46	66.37 ± 2.37	60.42 ± 4.84	55.62 ± 3.42
**AST (SGOT) (U/l)**	143.14 ± 5.26	156.27 ± 5.46	152.27 ± 6.27	153.82 ± 3.15	151.34 ± 6.89	139.18 ± 4.16	149.32 ± 5.37	146.72 ± 5.19
**ALP (U/l)**	251.61± 8.72	282.35 ± 6.49	280.34 ± 11.73	284.23 ± 7.49	271.47 ± 9.94	275.38 ± 8.17	273.56 ± 10.17	270.13 ± 6.48
**Total bilirubin (mg/dl)**	0.13 ± 0.03	0.17 ± 0.02	0.18 ± 0.03	0.17 ± 0.02	0.12 ± 0.01	0.15 ± 0.04	0.14 ± 0.03	0.15 ± 0.04
**Total cholesterol (mg/dl)**	98.76 ± 2.14	94.37 ± 1.49	91.42 ± 1.28	90.45 ± 2.94	99.52 ± 3.46	95.37 ± 4.81	92.29 ± 2.19	90.73 ± 0.73
**Triglyceride (mg/dl)**	57.68 ± 3.46	62.78 ± 3.18	61.27 ± 2.14	60.39 ± 1.21	59.63 ± 4.18	61.42 ± 0.89	60.13 ± 0.75	58.14 ± 0.98

Values expressed as ± SEM, one-way ANOVA followed by Dunnett’s test (*n*=5 animals/group). Differences between groups were considered to be significant when *P*<0.05.

Subacute toxicity protocols provide vital information regarding dosage regimens, target organ toxicity and recognises observable adverse effect which may affect lifespan of animals involved in experiment. In current investigation, effect of EECLS were evaluated in Wistar rats at doses of 250, 500 and 1000 mg/kg for 28 days. Change in body weight is an imperative indicator for general health status of animal. After repeated administration of extract for 28 days, all treatment group animals displayed a normal increment in body weight. This increase in body weight of animal during study attributed to significant increment in food and water intake (Figure 3). Therefore, it can be stated that extract did not interfere with the regular metabolism of treatment group rats [20,21].

The weight of vital organs such as lungs, liver, kidney, heart and spleen indicated no significant changes after administration of EECLS at subacute doses.

The haematopoietic system is most sensitive targets for toxic compounds and become significant index of physiological and pathological status in animal and human. As blood is the primary channel for transportation of nutrient and other foreign substances, assessment of haematological parameter can used to evaluate extent of detrimental effect of foreign compounds. In the present investigation, the haematological parameters were within the reference range for rat indicating that plant extract does not possess toxic compounds which can cause any abnormalities [22].

In safety evaluation, biochemical parameters have crucial role as they act as marker due to their response to sign and symptoms produced by toxicants. To assess the toxic effects produced by extract, estimation of hepatic and renal function is important. Liver plays pivotal role to remove foreign toxicants from body by metabolising them. Damage to the liver can be recognised by increase in levels of transaminase activity (ALT, AST and ALP) in blood. No significant changes were observed in serum levels of ALT, AST and ALP implying that extract exert protective action on liver [23]. Similarly, this result validates that of Bezabang et al. (2018) which displayed oral administration of *C. lanatus* seed extract protects liver significantly and prevent severe hepatic damage [24]. Also, histpathological analysis of the liver cells specified no lesions observed in treated animals indicating hepatoprotective action of extract.

Similar to liver, kidney also plays pivotal role in preserving stable state of the body by excreting metabolised waste products. Prolonged ingestion of extract might damage renal tubules of kidney and leads to nephrotoxicity. To estimate normal kidney function, we analysed serum urea and creatinine levels after administration of extract for 28 days. No changes in serum levels of urea, uric acid and creatinine was observed in treated rats when compared with normal control [25].

Different studies demonstrated the connection between dyslipidaemia and chronic disease progressions such as diabetes, hypertension etc. [26]. Decreased levels of serum glucose and total cholesterol levels implied that EECLS is useful in treatment of hyperglycaemia and hypercholesterolemia.

Histopathological evaluation of liver and kidney showed that EECLS not affected normal structural and appearance of treated rats when compared with normal control. The biochemical parameters (urea, uric acid, ALP, AST) of the study also supported histopathological results as they designate normal functioning of liver and kidney (Figures 6A,C and 7A,C).

## Conclusion

The current investigation shows that the oral dose upto 2000 mg/kg of EECLS is safe and did not display any treatment associated sign of toxicity or mortality in Wistar rats. Also, EECLS did not produce any adverse consequence on behaviour and gross pathology of treated animal. Therefore, the LD_50_ of the ethanol extract of *C. lanatus* seed was greater than 2000 mg/kg and included in category 5 as described by OECD guidelines. During 28 days repeated administration of EECLS, biochemical, haematological, behavioural and histological parameters were within safe limit for 250, 500 and 1000 mg/kg BW dose when compared with untreated control group. Also, no significant changes were observed in body weight, food and water intake of treated group animals. These results advocate that EECLS is safe to be used in assessment of pharmacological activity. As per those results the NOAEL of EECLS was at a dose of 1000 mg/kg in rats during the subacute toxicity study which provides basis for clinical use of *C. lanatus* seed extract.

## Data Availability

Data will be made available on request.

## Abbreviations

ALP: alkaline phosphatase
ALT: alanine aminotransferase
AST: aspartate aminotransferase
BW: Body weight
EECLS: ethanolic extract of *C. lanatus* seed
LD 50: Lethal dose causes the death of 50% (one half) of a group of test animals.
NOAEL: no observed adverse effect level
OECD: Organization for Economic Cooperation and Development
PL: platelet
